# Recovery trajectories in common musculoskeletal complaints by diagnosis contra prognostic phenotypes

**DOI:** 10.1186/s12891-021-04332-3

**Published:** 2021-05-19

**Authors:** Lene Aasdahl, Fredrik Granviken, Ingebrigt Meisingset, Astrid Woodhouse, Kari Anne I. Evensen, Ottar Vasseljen

**Affiliations:** 1 Department of Public Health and Nursing, Norwegian University of Science and Technology (NTNU), Postboks 8905, MTFS, 7491 Trondheim, Norway; 2 Unicare Helsefort Rehabilitation Centre, Rissa, Norway; 3 Department of Physical Medicine and Rehabilitation, St.Olavs Hospital, Trondheim, Norway; 4 Department of Clinical and Molecular Medicine, NTNU, Trondheim, Norway; 5 Department of Physiotherapy, Oslo Metropolitan University, Oslo, Norway; 6 Unit for Physiotherapy Services, Trondheim Municipality, Trondheim, Norway

**Keywords:** Musculoskeletal pain, Prognosis, Biopsychosocial, Subgrouping

## Abstract

**Background:**

There are large variations in symptoms and prognostic factors among patients sharing the same musculoskeletal (MSK) diagnosis, making traditional diagnostic labelling not very helpful in informing treatment or prognosis. Recently, we identified five MSK phenotypes across common MSK pain locations through latent class analysis (LCA). The aim of this study was to explore the one-year recovery trajectories for pain and functional limitations in the phenotypes and describe these in relation to the course of traditional diagnostic MSK groups.

**Methods:**

We conducted a longitudinal observational study of 147 patients with neck, back, shoulder or complex pain in primary health care physiotherapy. Data on pain intensity and function were collected at baseline (week 0) and 1, 2, 3, 4, 6, 8, 12, 26 and 52 weeks of follow up using web-based questionnaires and mobile text messages. Recovery trajectories were described separately for the traditional diagnostic MSK groups based on pain location and the same patients categorized in phenotype groups based on prognostic factors shared among the MSK diagnostic groups.

**Results:**

There was a general improvement in function throughout the year of follow-up for the MSK groups, while there was a more modest decrease for pain intensity. The MSK diagnoses were dispersed across all five phenotypes, where the phenotypes showed clearly different trajectories for recovery and course of symptoms over 12 months follow-up. This variation was not captured by the single trajectory for site specific MSK diagnoses.

**Conclusion:**

Prognostic subgrouping revealed more diverse patterns in pain and function recovery over 1 year than observed in the same patients classified by traditional diagnostic groups and may better reflect the diversity in recovery of common MSK disorders.

**Supplementary Information:**

The online version contains supplementary material available at 10.1186/s12891-021-04332-3.

## Background

It is generally accepted that the majority of patients with common musculoskeletal (MSK) pain disorders have non-specific symptoms without clear pathogenesis [1, 2]. Traditionally, non-specific MSK pain complaints are diagnostically labelled by the patient’s pain location, such as neck, shoulder, or low back pain. Despite large variations in symptoms, patient characteristics and prognostic factors among patients within the same diagnostic groups, treatment guidelines are launched to fit all [3]. Thus, it is not surprising that interventions for non-specific MSK pain complaints either lack evidence to support their use or at best have modest or only short-term effects [4–6]. In fact, effects may not be markedly different from the natural course of symptoms [7].

The multiplicity of biological, psychological, and social factors often seen in patients with MSK complaints challenges the idea of selecting treatment based on diagnosis or site of pain alone [8]. Traditional MSK diagnostic labelling by pain location does not reflect the heterogeneity and multiplicity of symptoms often seen in these patients, and provide limited guidance in differentiating patients and inform clinical management, also within the same diagnostic group. Secondary analyses of individual patient data from seven randomized controlled trials investigating a range of interventions across different regional MSK pain complaints showed similar patterns of improvement in pain and function regardless of pain location, whereas the magnitude of improvement varied by pain location [9]. Importantly, this variation was explained by prognostic factors, such as age, type of work (manual vs non-manual), pain duration, mood (anxiety and depression), and widespread pain [9]. Labelling patients and targeting treatment on prognostic factors rather than the location of pain, i.e., back pain, is supported by a study of Hill et al. [10], where treatment specifically targeting prognostic factors proved superior to usual care in improving primary care efficiency [10]. On this background, leading researchers have argued to focus on prognostic factors rather than diagnostic accuracy to improve clinical practice and patient outcome [8, 11]. Where diagnosis focuses on past and current status, prognosis relates to future events and outcome and is thus more suited to inform course of symptoms, recovery and anticipated outcome. This information is not explicitly revealed when patients are characterized solely by their site of pain. Although clinicians may use a more comprehensive approach to understand the patient’s ailments and plan treatment, the clinical reasoning and diagnostic work-up is not reflected in diagnostic labelling used for reimbursement, coding in medical records, and even in referrals to other stakeholders. In research literature MSK diagnosis by pain location prevails in communicating the results (low back pain, neck pain etc.). Thus, how patients are labelled matters. We are not aware of any studies of MSK pain patients that have compared prognostic trajectories with patients grouped by their prognostic capacity at baseline as opposed to their current pain location (MSK diagnosis).

We recently identified five MSK phenotypes across common non-specific MSK complaints based on generic prognostic factors in MSK pain conditions [12]. The phenotypes were identified by latent class analysis (LCA) and based on 11 prognostic factors covering all aspects of the biopsychosocial domain. The five phenotypes were clearly distinguished by level of symptoms, with different impact of the 11 prognostic factors on the phenotypes [12]. The five phenotype classes identified by the LCA algorithm were characterized with low symptoms scores across the biopsychosocial domain for class 1 and 2. Class 3 and 4 were more affected on most domains, but where class 3 showed higher fear avoidance and class 4 higher mental distress, while class 5 showed high levels of severity across all biopsychosocial domains. The purpose of this study within primary physiotherapy care was to explore the clinical course and recovery trajectories for pain and functional limitations over 1 year when the patients were labelled by phenotype (prognostic factors) versus their MSK diagnosis (pain location).

## Methods

### Study design, setting and participants

This longitudinal study used data from a large observational study in Norwegian primary health care physiotherapy (FYSIOPRIM) designed to describe patients, treatment and outcome in the sector as a whole [13]. For the current study we used data collected in this project from March to December 2017 in Trondheim Municipality. At the first consultation to the physiotherapist patients were asked if they could be contacted by a researcher for participation in the project. Inclusion criteria were patients seeking physiotherapy for complaints in the neck, shoulder, low back or had complex pain as their main problem. Complex pain was based on the judgement of the physiotherapist and comprised both multisite pain and complex pain problems (in terms of clusters of physical and/or mental conditions that complicated setting a main pain diagnosis). The physiotherapists categorized the patients’ main problem during the consultation. The main problem corresponded to the four site-specific MSK pain diagnoses neck, shoulder, low back, and complex pain. Exclusion criteria were rheumatoid arthritis, neurological conditions (e.g. stroke and multiple sclerosis), fractures, traumatic injuries, pre- or postoperative patients, pregnancy related disorders, other specific diagnoses (e.g. frozen shoulder and chronic obstructive pulmonary disease) and poor comprehension of the Norwegian language. Patients received “usual care physiotherapy”, where the physiotherapists decided the content and number of treatments. The study was approved by the Regional committee for Medical and Health Research Ethics in South East Norway (No.: 2013/2030).

### Data collection

Data was collected at 10 time-points: baseline (week 0, equivalent to first consultation) and at 1, 2, 3, 4, 6, 8, 12, 26 and 52 weeks after baseline. Participants answered web-based questionnaires at baseline (week 0), 12, 26 and 52 weeks, while questions were sent by mobile text messages (SMS) at weeks 1, 2, 3, 4, 6 and 8. In addition, they answered some questions in collaboration with the physiotherapist at baseline and 12 weeks of follow-up. The patients received a reminder if they did not fill out the questionnaires. The software for the SMS data collection was provided by SMS-Track ApS, Denmark (www.sms-track.com).

### Questionnaires

The baseline questionnaire encompassed background information on age, sex, and education. Education was categorized as primary school (or less), high school, up to 4 years of higher education and more than 4 years of higher education.

Pain intensity was assessed with the question “How would you rate the pain that you have had during the past week”, rated on a numeric rating scale from 0 (no pain) to 10 (as bad as it could be) [14]. Function was assessed by the Patient Specific Functional Scale (PSFS) [15], which asked the participants to identify activities that they are unable to perform or have difficulty performing due to their problem. They were then asked to rate the difficulty on a scale from 0 (unable to perform) to 10 (able to perform at prior level). The activity was defined together with the physiotherapist at baseline, and at subsequent questionnaires the participant was asked to rate the same activity. Pain intensity and PSFS was assessed at all time-points.

Other variables registered by questionnaires at baseline was number of pain locations (up to 10 marked on a body chart) [16], pain duration (10 response options collapsed into up to 4 weeks, between 4 and 12 weeks, between 26 and 52 weeks, and over 52 weeks) [14], and whether the pain was continuous or not. Recovery expectations were assessed by the item “In your view, how large is the risk that your current pain may become persistent?” [14], rated on a 0–10 numeric rating scale (0 no risk; 10 very high risk). Pain self-efficacy was assessed by two items from the Pain self-efficacy questionnaire (“I can do some form of work, despite pain (work includes housework and paid/unpaid work)”, and “I can live a normal life, despite pain”) [17]. Both items scored from 0 (not at all) to 6 (completely confident), and the items were summed. Mental distress was measured by the Hopkins Symptom Check List 10-item version (HSCL-10), with scores ranging from 1 (low) to 4 (high) [18, 19]. Fear-Avoidance was measured on a numeric rating scale 0–10 with one question (“I should not do my normal activities or work with my present pain”) [14]. The item “What is your current work ability compared with the lifetime best?” measured work ability, and was scored on a numeric rating scale 0–10 (10 best) [20]. Daily activity level was assessed by the question “Due to pain or complaints, how much have your activities of daily life been reduced?” with response options “not reduced”, “slightly reduced”, “quite reduced”, and “very reduced”. A single item from 15D was used to assess sleep problems (scoring options “I’m able to sleep normally”, “I have slight problems with sleeping”, “I have moderate problems with sleeping”, “I have great problems with sleeping”, “I suffer severe sleeplessness”) [21].

### Statistical analyses

Sample size calculation was not performed for this study as we utilized previously collected data to explore and describe the clinical course and prognosis.

In addition to the MSK diagnosis groups, participants were classified into five different phenotypes based on LCA; developed and validated in a previous study [12]. To derive the phenotypes 11 different variables (pain intensity, number of pain locations, frequency of pain, pain duration, recovery expectations, pain self-efficacy, mental distress, fear avoidance, sleep problems, work ability, and daily activity level) and three covariates (age in years, sex, and education) were used [12]. The participants in the present study were classified using the same algorithm as in the previous study. This procedure is described in detail in Meisingset et al. [12].

Linear mixed models were used to estimate mean scores for pain and function (PSFS) at the different time-points. The models were adjusted for age, sex, and education, and stratified for MSK diagnosis and LCA phenotypes. These models were also used to estimate change in pain and PSFS scores at the different time-points. Percentage of participants that recovered was calculated at the different time-points, both for the diagnosis groups and the LCA phenotypes. Recovery was defined as a pain score of 3 or less and/or a PSFS score of 8 or more (both scored on a scale from 0 to 10).

The analyses were done using STATA 14 (StataCorp. 2015. Stata Statistical Software: Release 14. College Station, TX: StataCorp LP).

## Results

**Fig. 1:**
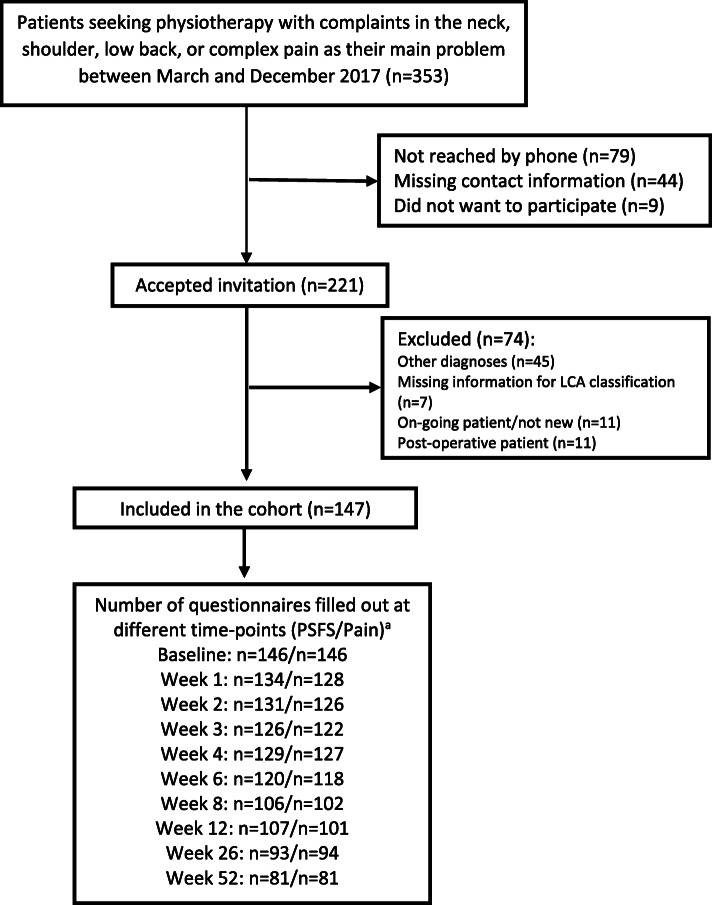
Flow of participants through the trial. PSFS = Patient Specific Functional Scale. LCA: latent class analysis.^a^ Questionnaires at baseline, 12, 26 and 52 weeks were filled out on a tablet, the other by text message

Recruitment of study participants and follow-up rates are given i Fig. 1. We recruited 147 patients at baseline. PSFS data were available for 88, 73, 63 and 55% at 4, 12, 26 and 52 weeks, respectively, with slightly lower rates for pain intensity. The participants’ mean age was 45 (SD 15) years, the majority were women (73%), mean pain intensity was moderate (5.0, SD 2.2) and participants had a considerably reduced function (mean PSFS score 3.8, SD 2.1) (Table 1). Most participants (74%) had chronic pain lasting more than 3 months and only 6% had pain lasting less than 4 weeks.

**Table 1:** Baseline characteristics of included patients

	Total cohort (*n* = 147)
**Female** n %	107 (73)
**Age** mean (SD)	45 (15)
**Education** n (%)	
Primary school or less	3 (2)
High school	55 (37)
Up to 4 years of higher education	55 (37)
More than 4 years of higher education	32 (22)
Unknown	2 (1)
**Pain duration n (%)**^a^	
0–4 weeks	9 (6)
4–11 weeks	29 (20)
3–6 months	26 (18)
6–12 months	18 (13)
Over 1 year	61 (43)
**Pain intensity** (0–10)^b^ mean (SD)	5.0 (2.2)
**PSFS** (0–10)^b,c^ mean (SD)	3.8 (2.1)
**Main problem area** n (%)	
Neck	40 (27)
Shoulder	48 (33)
Back	31 (21)
Complex	28 (19)
**LCA classes** n (%)	
Class 1	21 (14)
Class 2	49 (33)
Class 3	35 (24)
Class 4	26 (18)
Class 5	16 (11)

^a^*n* = 143

^b^*n* = 146

^c^*PSFS* Patient Specific Functional Scale. Scored on a 11-point scale from 0 (unable to perform) to 10 (able to perform at prior level)

### Course of pain and function over one year

**Fig. 2:**
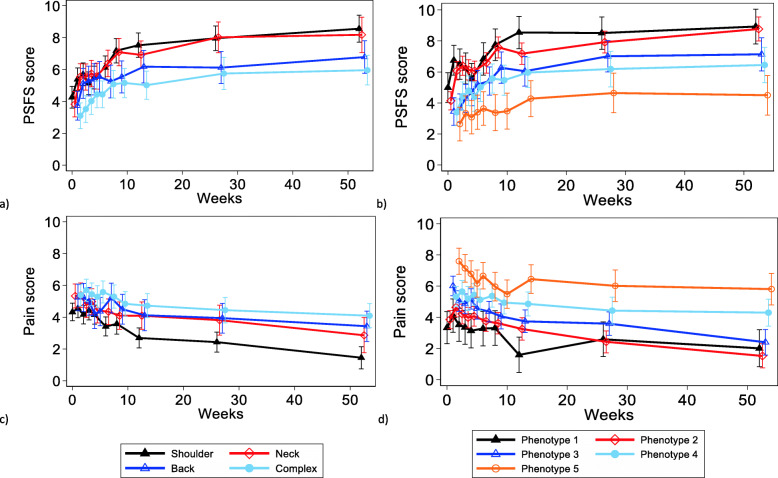
Function (Patient Specific Functional Scale; PSFS) and pain trajectories over the 52-week follow-up period for the MSK diagnoses groups (**a and c**) and the LCA phenotype classes (**b and d**). The estimated means are based on linear mixed models adjusted for age, sex and education

Recovery trajectories for the MSK diagnoses and phenotypes based on the linear mixed models are shown in Fig. 2. The four MSK groups showed similar improvement patterns in function. The shoulder and neck pain patients attained greater functional improvement than the low back and complex patients over the one-year follow-up period (Fig. 2a). A true and stable mean improvement of two points or more on the PSFS scale, defined by the lower limits of the 95% confidence interval, was attained at 8 weeks for shoulder and neck pain patients, while the equivalent level was not reached at all over the 52-week follow-up for the low back and complex pain patients (Online Table 1). The mean change on the PSFS scale by 8 weeks was 2.9 (95% CI 2.1–3.7) and 3.3 (95% CI 2.4–4.2) for the shoulder and neck pain patients, respectively (Online Table 1). Except for the shoulder and to some degree the neck pain patients, improvements in pain intensity were small within the other two MSK groups over 52 weeks (Fig. 2c and Online Table 2). Changes in pain intensity of two points or more by the same criteria as for PSFS above were not reached in any of the MSK groups during the 52-weeks follow-up, except for the shoulder pain patients at 52 weeks (− 2.9, 95% CI − 3.6 to − 2.2) (Online Table 2).

**Table 2:** Scores on the variables used to derive the LCA (latent class analysis) phenotype classes for the different musculoskeletal diagnosis groups

	Pain area			
	Neck	Shoulder	Back	Complex
**Pain variables**				
Pain intensity (0–10), mean (SD)	5.3 (2.3)	4.3 (2.1)	5.3 (2.2)	5.4 (2.1)
Number of pain sites (0–10), mean (SD)	3.7 (2.1)	2.4 (1.4)	3.2 (2.5)	5.6 (2.6)
Continuous pain^a^, n (%)	22 (58)	21 (44)	13 (42)	14 (50)
Pain duration, n (%)				
< 3 months	13 (35)	16 (33)	5 (17)	4 (14)
3- < 12 months	11 (30)	17 (35)	12 (40)	4 (14)
≥ 12 months	13 (35)	15 (31)	13 (43)	20 (71)
**Beliefs and thoughts**				
Recovery expectations^b^ (0–10), mean (SD)	5.6 (3.2)	4.5 (2.5)	6.4 (2.3)	7.0 (2.4)
Pain self-efficacy^c^ (0–12),mean (SD)	8.7 (2.5)	10.0 (2.5)	9.5 (2.6)	7.6 (2.7)
**Psychological**				
Mental distress^d^ (1–4), mean (SD)	1.9 (0.5)	1.5 (0.4)	1.7 (0.5)	2.3 (0.6)
Fear avoidance^e^ (0–10), mean (SD)	4.3 (3.4)	3.3 (3.4)	3.1 (2.9)	4.4 (3.3)
**Activity and lifestyle**				
Work ability^f^ (0–10),mean (SD)	5.6 (2.8)	7.0 (2.6)	6.2 (3.0)	3.8 (2.4)
Daily activity level^g^, n (%)				
Very much reduced	7 (18)	1 (2)	4 (13)	5 (18)
Quite reduced	10 (26)	12 (26)	12 (42)	15 (54)
Slightly reduced	20 (53)	25 (53)	10 (32)	7 (25)
Not reduced	1 (3)	9 (19)	4 (13)	1 (4)
Sleep^h^, n (%)				
No problem	5 (13)	14 (29)	11 (37)	3 (11)
Slight problems	16 (41)	24 (50)	12 (40)	10 (36)
Moderate problems	13 (33)	9 (19)	4 (13)	12 (43)
Great/severe problems	5 (13)	1 (2)	3 (10)	3 (11)

^a^Continuous pain: “Is the pain was continuous?”

^b^Recovery expectation: “In your view, how large is the risk that your current pain may become persistent?” (0 = no risk; 10 = very large risk)

^c^Pain self-efficacy: Two questions: 1) “I can do some form of work, despite pain (work includes housework and pain/unpaid work”, and 2) “I can live a normal lifestyle, despite pain”. Response options ranging from 0 (not at all) to 6 (completely confident) on both questions. Response options added together, ranging from 0 to 12 (higher score indicate higher levels of self-efficacy)

^d^Mental distress: The Hopkins Symptom Check List-10 item (HSCL-10)

^e^Fear avoidance: «I should not do my normal activities or work with my present pain?” (0 = completely disagree; 10 completely agree)

^f^Work ability: «What is your current work ability compared with the lifetime best?” (10 = best)

^g^“Due to pain or complaints, how much reduced is your activities of daily life?”

^h“^Which alternative best describes your present sleeping status”

**Fig. 3:**
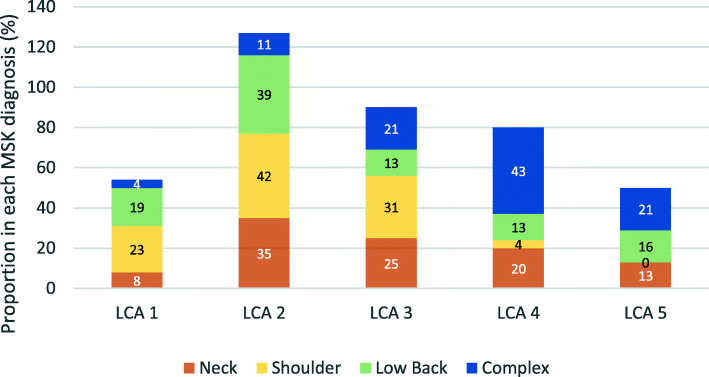
Distribution of musculoskeletal (MSK) diagnosis across the latent class analysis (LCA) phenotype classes. The number represents percentage of participants with each diagnosis in an LCA class out of all participants with that diagnosis (i.e. 100% across the columns)

The courses of pain intensity and function showed different trajectories for the five LCA phenotypes and appeared visually more dispersed across the phenotype classes than the MSK diagnoses (Fig. 2b, d). The 11 prognostic variables used to model the five LCA classes are shown for each MSK diagnosis in Table 2. Scores on the prognostic variables were similar across the MSK groups except for the complex pain patients who were characterized by higher somatic and mental symptom pressure, clearly reduced work ability and more sleep problems. Applying the LCA on the patients in the MSK diagnostic groups in order to identify the prognostic phenotype group of best fit, showed that the patients were spread across all five phenotypes (Fig. 3). The only MSK diagnostic group that was not represented in all five phenotypes was shoulder pain, which was represented only in phenotype 1–4.

### Recovery

**Fig. 4:**
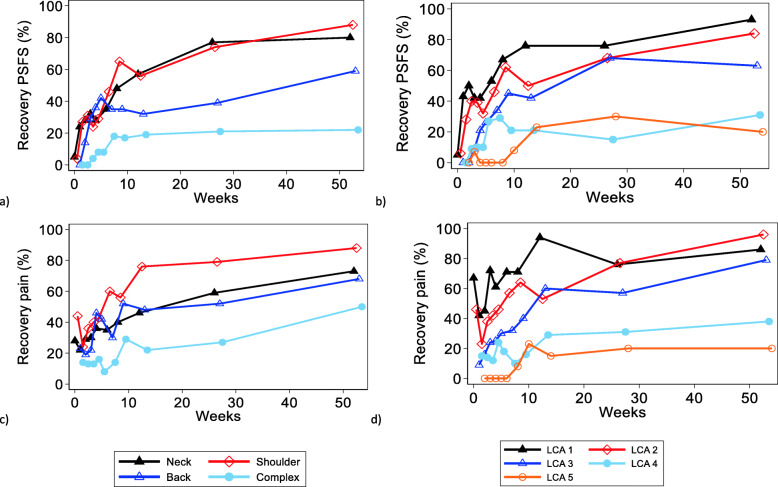
Percentage of participants with recovery based on their Patient Specific Functional Scale (PSFS) and pain scores during 52 weeks follow-up period. Recovery was defined as pain score of 3 or less and/or PSFS score 8 or higher. Figure (**a**) shows the PSFS scores for the MSK diagnosis groups and (**b**) the LCA phenotype classes. Figure (**c**) shows the pain scores for the MSK diagnosis groups and (**d**) the LCA phenotype classes

Figure 4 shows the recovery trajectories for the four MSK groups (Fig. 4a, c) and the five phenotypes (Fig. 4b, d). The proportions of patients functionally recovered by 12 weeks were 19, 32, 56 and 57% in the complex, low back, shoulder, and neck groups, respectively. For pain at 12 weeks, 22, 48, 76 and 46% of the patients were recovered in the complex, low back, shoulder, and neck groups, respectively.

Recovery in the phenotype classes showed an orderly and clearly decreasing proportion of recovered patients from LCA class 1 to 5 (Fig. 4). Patients in LCA classes 1–3 showed steady, increasing recovery rates for both pain and function, where changes mostly happened the first 12 weeks, and 2/3 or more reached recovery by 52 weeks. Recovery rates for pain and function in LCA classes 4–5 were modest, were only about 1/3 recovered over the 52-weeks period.

### Participants with missing data

Participants with missing information at 52 weeks of follow-up were somewhat younger (mean age 43 [SD 15] vs 47 [SD 16]) and more likely to be men (33% vs 22%) than those responding. Baseline scores for pain intensity and function were similar for those responding and those not responding (pain 4.9 [SD 2.2] vs. 5.1 [SD 2.3]; PSFS 3.8 [SD 2.2] vs 3.8 [SD 2.1]). Pain duration, education level and distribution between the LCA classes were also similar between those responding and not, while the diagnoses varied a bit. Participants not responding had to a larger degree neck pain (38% vs 19%) and less back pain (14% vs 27%) and complex pain (15% vs 22%) than those responding.

## Discussion

We explored one-year clinical course and recovery trajectories for function and pain in patients seeking primary care physiotherapy for common MSK complaints. All patients were classified by their phenotype (prognostic factors) as well as their MSK diagnosis (pain location). A general improvement in function was observed throughout the year of follow-up for the MSK groups, with smaller changes in pain intensity. Noticeably, we found that within each MSK diagnostic group (neck, shoulder, low back and complex pain), the patients were spread across all phenotype classes. Expressing course and recovery trajectories by diagnosis did not reflect the actual variation in prognosis within the diagnostic groups. The phenotype classes showed a clearly decreasing rate of recovery from class 1 to class 5.

The variation in improvement between the prognostic subgroups is in line with previous research demonstrating large differences in recovery patterns within the same diagnostic group, like low back pain and neck pain [22–24]. Relative to patients with the neck, shoulder, and low back pain, patients with complex pain had more pain locations, longer pain duration, less recovery expectations, more mental distress, less work ability, lower daily activity levels and more sleep problems. Most of these factors are known as negative predictors and the findings comply with previous research [25]. The observed improvements occurred mostly during the first 12 weeks of follow-up, both for the diagnostic groups and the phenotypes, which is in line with previous research [7, 26].

Patients in the MSK groups were distributed throughout all five phenotype classes, except for shoulder pain patients who were not represented in phenotype class 5. The MSK diagnoses neck, shoulder and low back pain showed similar dispersion across the five phenotype classes, although the majority were clustered in the lower three phenotype classes. Patients with complex pain were mainly clustered in phenotype class 4 and 5, with low recovery rates, but surprisingly, 15% of the patients were classified in phenotype class 1 and 2 with the highest recovery rates. Contrary, while most neck pain patients were classified in the lower three phenotype classes with the highest recovery rates, 33% of the patients were classified in phenotype class 4 and 5 with the lowest recovery rates. This underscores that large variations in recovery rates exist among individuals within the same MSK group, and that on the individual level, phenotyping will provide higher precision on likely course. This information is important in clinical practice when setting up achievable goals, realistic timeframes and addressing the patients’ expectations. For patients with phenotypes with a less positive course therapists could, instead of focusing on pain location and intensity only, include a broader aspect of factors of importance for recovery early on, e.g., active coping for restoring function and activity despite pain. In addition, information about phenotypes could inform the clinician about expected amount of resources needed for treatment.

In compliance with previous studies [12, 27–29], we observed similar distributions of prognostic factors across the site-specific MSK groups neck, shoulder, and low back pain, as well as for the clinical course of pain and function. However, there were large variations within each site-specific group in the individuals’ scores on the prognostic factors, also for patients with complex pain (Table 2). Individual variations in scores on prognostic variables are not communicated well by MSK diagnoses, which merely define the location of the pain. Subdividing patients into prognostic phenotypes will reveal variations in clinical course and recovery not apparent in equivalent trajectories by MSK diagnosis. Thus, when it comes to estimating prognosis and deciding management, the patient heterogeneity within traditional MSK diagnostic groups can be reduced by emphasizing on prognostic phenotypes rather than diagnoses relying on location of the pain.

Drawing attention to prognostic factors in musculoskeletal pain research is not new. We acknowledge that prior research has pointed out several important prognostic factors in MSK pain conditions [30–32], and that they are better prognostic indicators than MSK diagnosis [8]. There are several examples of initiatives to move away from traditional pain location diagnostics, e.g., by launching clinical prediction rules [33–35], prediction models [36, 37], and clustering in risk strata [10]. This development is however still in the molding and progressing slowly [38, 39], and introduction of prediction models in the clinic is not straight forward [40]. Our approach adds to this literature by arching the whole biopsychosocial domain of known prognostic factors across common MSK pain conditions. By their construction, it is reasonable to assume that the phenotype groups must be more homogeneous in terms of prognosis. Stratifying patients into prognostic rather than diagnostic groups should thus increase the accuracy for estimating patient prognosis in clinical settings and providing more individually adapted estimates. For MSK patients, it is extremely challenging even for the most competent clinician in their encounter with individual patients to estimate the prognostic impact of a range of variables and their numerous combinations. Systematically collecting patients’ responses to known prognostic factors and categorizing these patients into prognostic phenotypes will raise reliability among clinicians, improve accuracy of estimating prognosis for individual patients, as well as patient management and decision-making in clinical settings.

For interpretation of the different phenotypes we refer to our previous study [12]. In summary, phenotype 1 and 2 were characterized by the lowest scores across all biopsychosocial domains, but phenotype 2 had somewhat higher levels of symptoms across the domains. Phenotype 3 and 4 were more affected in all domains compared to phenotype 1 and 2. The main differences between phenotype 3 and 4 were the opposite scoring pattern within the psychological domain (higher fear avoidance and lower mental distress in phenotype 3 and vice versa for phenotype 4), and worse symptoms in the pain domain (longer pain duration and more pain locations) in phenotype 4. Phenotype 5 was characterized by worse symptoms across all domains, especially in the domain “pain” and “activity and lifestyle. Overall, higher phenotype affiliation tended to be associated with more obesity and lower education.

One strength of this study was the repeated collection of data for pain and function from baseline and up to 52 weeks. There are also some limitations. Firstly, function is difficult to measure. A strength of the PSFS is that patients could report on functional activities that were important for them to improve. But this also means that the type of functional activities reported varied between patients, and that the scores could be similar between patients despite very different functional activities. Nevertheless, the PSFS have shown to be a sensitive measure of change [41]. Furthermore, change in pain should be interpreted with caution for the phenotype classes, as pain was one of the variables used to derive the LCA classes, i.e. potential problems with floor effects and regression towards the mean. Another limitation was the relatively small sample size and missing data. Missing data for the first five follow-up time-points was however marginal, besides data were analyzed with linear mixed models which accounts for all available data. There was no information on treatment modalities in this study or what information the physiotherapist based their treatment strategy on. Thus, treatment recommendations for the different subgroups cannot be extracted.

## Conclusions

Classifying MSK patients in prognostic phenotypes rather than traditional diagnostic groups was superior in reflecting patient variation in clinical course and recovery among patients with common MSK pain complaints. For clinical practice it may be more informative to subclassify patients according to prognostic factors rather than site-specific MSK diagnostic groups, as prognostic phenotyping provided more precise information of individual patients’ potential for recovery. Future studies should evaluate stratified treatment based on the prognostic phenotype classes.

## Supplementary Information

**Additional file 1:** **Online Table 1.** Estimated mean change in Patient specific functional scale (PSFS) from baseline. **Online Table 2.** Estimated mean change in pain from baseline.

## Data Availability

The datasets generated and/or analyzed during the current study are not publicly available due to permission has not been applied for from neither the participants nor the Ethical Committee but might be available from the corresponding author on reasonable request.

## Abbreviations

HSCL-10: Hopkins Symptom Check List 10-item version
LCA: Latent class analysis
MSK: Musculoskeletal
PSFS: Patient Specific Functional Scale
