# Correction to: Recovery trajectories in common musculoskeletal complaints by diagnosis contra prognostic phenotypes

**DOI:** 10.1186/s12891-021-04428-w

**Published:** 2021-06-25

**Authors:** Lene Aasdahl, Fredrik Granviken, Ingebrigt Meisingset, Astrid Woodhouse, Kari Anne I. Evensen, Ottar Vasseljen

**Affiliations:** 1 Department of Public Health and Nursing, Norwegian University of Science and Technology (NTNU), Postboks 8905, MTFS, 7491 Trondheim, Norway; 2 Unicare Helsefort Rehabilitation Centre, Rissa, Norway; 3 Department of Physical Medicine and Rehabilitation, St.Olavs Hospital, Trondheim, Norway; 4 Department of Clinical and Molecular Medicine, NTNU, Trondheim, Norway; 5 Department of Physiotherapy, Oslo Metropolitan University, Oslo, Norway; 6 Unit for Physiotherapy Services, Trondheim Municipality, Trondheim, Norway

**Correction to: BMC Musculoskelet Disord 22, 455 (2021)**

**https://doi.org/10.1186/s12891-021-04332-3**

Following the publication of the original article [1] the authors noticed that the Online Tables 1 and 2 (supplementary) were missing in the proof. The citations were linked to the normal tables.

The original article [1] has been updated.

## Supplementary Information

**Additional file 1: Online Table 1.** Estimated mean change in Patient specific functional scale (PSFS) from baseline. **Online Table 2.** Estimated mean change in pain from baseline.
