# Supplementary material for: Recovery trajectories in common musculoskeletal complaints by diagnosis contra prognostic phenotypes
Source: BMC Musculoskelet Disord. 2021 May 19;22:455. doi: 10.1186/s12891-021-04332-3 (PMC8132354; doi:10.1186/s12891-021-04332-3)
Supplement: Supplementary file 1 — Additional file 1: Online Table 1. Estimated mean change in Patient specific functional scale (PSFS) from baseline. Online Table 2. Estimated mean change in pain from baseline. [file 12891_2021_4332_MOESM1_ESM.docx]

**Online Table 1** Estimated mean change in Patient specific functional scale (PSFS) from baseline. Estimates are based on a linear mixed model adjusted for age, sex and education. The cells are colored in green where the lower limit of the 95% confidence interval is larger than two points.

|  | **Shoulder** | | | **Neck** | | | **Back** | | | **Complex** | | |
| --- | --- | --- | --- | --- | --- | --- | --- | --- | --- | --- | --- | --- |
| **Week** | **Estimated mean Δ** | **95 % CI** | **p** | **Estimated mean Δ** | **95 % CI** | **p** | **Estimated mean Δ** | **95 % CI** | **p** | **Estimated mean Δ** | **95 % CI** | **p** |
| **1** | 1.13 | 0.40-1.86 | 0.002 | 1.73 | 0.89-2.57 | <0.001 | 1.40 | 0.37-2.43 | 0.007 | 0.42 | -0.43-1.26 | 0.334 |
| **2** | 1.43 | 0.69-2.17 | <0.001 | 1.71 | 0.86-2.55 | <0.001 | 1.54 | 0.51-2.57 | 0.003 | 0.91 | 0.07-1.76 | 0.034 |
| **3** | 0.87 | 0.13-1.61 | 0.021 | 1.93 | 1.06-2.79 | <0.001 | 1.65 | 0.58-2.72 | 0.002 | 1.34 | 0.49-2.19 | 0.002 |
| **4** | 1.23 | 0.49-1.97 | 0.001 | 1.76 | 0.92-2.61 | <0.001 | 1.81 | 0.75-2.86 | 0.001 | 1.34 | 0.49-2.18 | 0.002 |
| **6** | 1.87 | 1.12-2.61 | <0.001 | 2.56 | 1.70-3.42 | <0.001 | 1.49 | 0.40-2.59 | 0.007 | 1.98 | 1.10-2.86 | <0.001 |
| **8** | 2.91 | 2.12-3.70 | <0.001 | 3.27 | 2.39-4.16 | <0.001 | 1.81 | 0.71-2.90 | 0.001 | 2.05 | 1.11-2.99 | <0.001 |
| **12** | 3.26 | 2.47-4.05 | <0.001 | 3.10 | 2.20-3.99 | <0.001 | 2.45 | 1.34-3.56 | <0.001 | 1.91 | 1.02-2.81 | <0.001 |
| **26** | 3.69 | 2.91-4.48 | <0.001 | 4.19 | 3.21-5.18 | <0.001 | 2.39 | 1.30-3.49 | <0.001 | 2.65 | 1.62-3.67 | <0.001 |
| **52** | 4.30 | 3.44-5.16 | <0.001 | 4.35 | 3.22-5.49 | <0.001 | 3.05 | 1.94-4.16 | <0.001 | 2.86 | 1.92-3.80 | <0.001 |

|  | **LCA 1** | | | **LCA 2** | | | **LCA 3** | | | **LCA 4** | | | **LCA 5** | | |
| --- | --- | --- | --- | --- | --- | --- | --- | --- | --- | --- | --- | --- | --- | --- | --- |
| **Week** | **Estimated mean Δ** | **95 % CI** | **p** | **Estimated mean Δ** | **95 % CI** | **p** | **Estimated mean Δ** | **95 % CI** | **p** | **Estimated mean Δ** | **95 % CI** | **p** | **Estimated mean Δ** | **95 % CI** | **p** |
| **1** | 1.76 | 0.64-2.88 | 0.002 | 1.94 | 1.22-2.65 | <0.001 | 0.21 | -0.69-1.12 | 0.642 | 1.04 | -0.02-2.10 | 0.054 | 0.69 | -0.53-1.92 | 0.267 |
| **2** | 1.51 | 0.37-2.64 | 0.009 | 2.26 | 1.52-2.99 | <0.001 | 0.85 | -0.4-1.73 | 0.061 | 1.39 | 0.30-2.48 | 0.013 | 0.44 | -0.74-1.62 | 0.467 |
| **3** | 1.23 | 0.08-2.39 | 0.037 | 2.00 | 1.25-2.76 | <0.001 | 1.27 | 0.38-2.16 | 0.005 | 1.00 | -0.8-2.07 | 0.069 | 0.76 | -0.44-1.96 | 0.215 |
| **4** | 0.55 | -0.61-1.70 | 0.352 | 1.93 | 1.19-2.67 | <0.001 | 1.74 | 0.84-2.64 | <0.001 | 1.62 | 0.56-2.68 | 0.003 | 1.00 | -0.18-2.18 | 0.097 |
| **6** | 1.85 | 0.65-3.04 | 0.002 | 2.45 | 1.69-3.21 | <0.001 | 1.96 | 1.06-2.85 | <0.001 | 2.18 | 1.10-3.25 | <0.001 | 0.73 | -0.53-1.98 | 0.256 |
| **8** | 2.75 | 1.58-3.93 | <0.001 | 3.45 | 2.67-4.23 | <0.001 | 2.82 | 1.82-3.82 | <0.001 | 2.07 | 0.96-3.18 | <0.001 | 0.83 | -0.42-2.09 | 0.194 |
| **12** | 3.55 | 2.36-4.75 | <0.001 | 3.04 | 2.24-3.84 | <0.001 | 2.59 | 1.64-3.55 | <0.001 | 2.57 | 1.46-3.68 | <0.001 | 1.65 | 0.39-2.90 | 0.010 |
| **26** | 3.51 | 2.32-4.71 | <0.001 | 3.78 | 2.98-4.59 | <0.001 | 3.56 | 2.56-4.56 | <0.001 | 2.79 | 1.53-4.05 | <0.001 | 2.00 | 0.64-3.37 | 0.004 |
| **52** | 3.93 | 2.67-5.20 | <0.001 | 4.63 | 3.77-5.50 | <0.001 | 3.69 | 2.63-4.74 | <0.001 | 3.05 | 1.79-4.31 | <0.001 | 1.86 | 0.49-3.22 | 0.008 |

**Online Table 2** Estimated mean change in pain from baseline. Estimates are based on a linear mixed model adjusted for age, gender and education. The cells are colored in green where the lower limit of the 95% confidence interval is larger than two points.

|  | **Shoulder** | | | **Neck** | | | **Back** | | | **Complex** | | |
| --- | --- | --- | --- | --- | --- | --- | --- | --- | --- | --- | --- | --- |
| **Week** | **Estimated mean Δ** | **95 % CI** | **p** | **Estimated mean Δ** | **95 % CI** | **p** | **Estimated mean Δ** | **95 % CI** | **p** | **Estimated mean Δ** | **95 % CI** | **p** |
| **1** | 0.22 | -0.41-0.85 | 0.492 | 0.04 | -0.83-0.91 | 0.925 | 0.00 | -1.01-1.01 | 0.996 | 0.32 | -0.35-0.99 | 0.347 |
| **2** | -0.17 | -0.79-0.46 | 0.602 | -0.57 | -1.46-0.32 | 0.210 | -0.24 | -1.24-0.76 | 0.637 | 0.07 | -0.61-0.74 | 0.850 |
| **3** | 0.09 | -0.55-0.72 | 0.786 | -0.36 | -1.26-0.55 | 0.439 | -1.04 | -2.08-0.00 | 0.050 | -0.09 | -0.75-0.57 | 0.783 |
| **4** | -0.17 | -0.81-0.46 | 0.596 | -0.91 | -1.79- -0.04 | 0.042 | -0.88 | -1.89-0.13 | 0.089 | 0.20 | -0.46-0.86 | 0.547 |
| **6** | -0.91 | -1.55- -0.28 | 0.005 | -0.99 | -1.89- -0.10 | 0.029 | -0.03 | -1.08-1.02 | 0.957 | -0.08 | -0.78-0.61 | 0.820 |
| **8** | -0.75 | -1.43- -0.07 | 0.031 | -1.25 | -2.17- -0.32 | 0.008 | -0.74 | -1.79-0.31 | 0.167 | -0.54 | -1.28-0.21 | 0.158 |
| **12** | -1.64 | -2.31- -0.97 | <0.001 | -1.24 | -2.19- -0.29 | 0.010 | -1.12 | -2.20- -0.03 | 0.043 | -0.65 | -1.38-0.08 | 0.081 |
| **26** | -1.89 | -2.56- -1.23 | <0.001 | -1.53 | -2.55- -0.50 | 0.004 | -1.30 | -2.35- -0.25 | 0.016 | -0.93 | -1.71- -0.16 | 0.019 |
| **52** | -2.88 | -3.61- -2.15 | <0.001 | -2.47 | -3.65- -1.29 | <0.001 | -1.81 | -2.88- -0.74 | 0.001 | -1.28 | -2.01- -0.55 | 0.001 |

|  | **LCA 1** | | | **LCA 2** | | | **LCA 3** | | | **LCA 4** | | | **LCA 5** | | |
| --- | --- | --- | --- | --- | --- | --- | --- | --- | --- | --- | --- | --- | --- | --- | --- |
| **Week** | **Estimated mean Δ** | **95 % CI** | **p** | **Estimated mean Δ** | **95 % CI** | **p** | **Estimated mean Δ** | **95 % CI** | **p** | **Estimated mean Δ** | **95 % CI** | **p** | **Estimated mean Δ** | **95 % CI** | **p** |
| **1** | 0.78 | -0.33-1.89 | 0.169 | 0.78 | 0.04-1.52 | 0.038 | -0.96 | -1.78- -0.15 | 0.020 | 0.33 | -0.50-1.16 | 0.439 | -0.46 | -1.47-0.56 | 0.378 |
| **2** | 0.18 | -0.91-1.28 | 0.744 | 0.36 | -0.40-1.12 | 0.354 | -1.15 | -1.95- -0.35 | 0.005 | -0.12 | -1.01-0.77 | 0.792 | -0.81 | -1.79-0.16 | 0.102 |
| **3** | 0.03 | -1.11-1.16 | 0.965 | 0.13 | -0.66-0.91 | 0.752 | -0.83 | -1.64- -0.03 | 0.043 | 0.12 | -0.71-0.95 | 0.778 | -1.43 | -2.42- -0.44 | 0.005 |
| **4** | -0.20 | -1.33-0.94 | 0.733 | 0.21 | -0.54-0.96 | 0.590 | -1.46 | -2.28- -0.64 | <0.001 | -0.20 | -1.02-0.63 | 0.641 | -0.94 | -1.91- 0.04 | 0.059 |
| **6** | -0.06 | -1.21-1.10 | 0.924 | -0.11 | -0.88-0.67 | 0.790 | -1.64 | -2.46- -0.83 | <0.001 | 0.05 | -0.80-0.89 | 0.909 | -1.63 | -2.67-0.60 | 0.002 |
| **8** | -0.02 | -1.17-1.13 | 0.970 | -0.23 | -0.04-0.57 | 0.568 | -1.95 | -2.88- -1.02 | <0.001 | -0.38 | -1.24-0.48 | 0.382 | -2.11 | -3.14- -1.07 | <0.001 |
| **12** | -1.74 | -2.91-0.57 | 0.004 | -0.61 | -1.44-0.22 | 0.152 | -2.27 | -3.13- -1.40 | <0.001 | -0.46 | -1.35-0.43 | 0.312 | -1.15 | -2.18- -0.11 | 0.030 |
| **26** | -0.74 | -1.89-0.41 | 0.206 | -1.43 | -2.25- -0.61 | 0.001 | -2.43 | -3.32- -1.54 | <0.001 | -0.90 | -1.88-0.08 | 0.072 | -1.58 | -2.70- -0.45 | 0.006 |
| **52** | -1.32 | -2.55- -0.09 | 0.034 | -2.33 | -3.21- - 1.46 | <0.001 | -3.61 | -4.56- -2.67 | <0.001 | -1.02 | -2.00- -0.05 | 0.039 | -1.79 | -2.92- -0.67 | 0.002 |
